# Suicide by Opioid: Exploring the Intentionality of the Act

**DOI:** 10.7759/cureus.18084

**Published:** 2021-09-18

**Authors:** Joseph Pergolizzi, Frank Breve, Peter Magnusson, Rohit Nalamasu, Jo Ann K LeQuang, Giustino Varrassi

**Affiliations:** 1 Cardiology, Native Cardio, Inc., Naples, USA; 2 Department of Pharmacy, Temple University, Philadelphia, USA; 3 Cardiology, Center of Research and Development Region Gävleborg, Uppsala University, Gävle, SWE; 4 Medicine, Cardiology Research Unit, Karolinska Institutet, Stockholm, SWE; 5 Department of Physical Medicine and Rehabilitation, University of Nebraska Medical Center, Omaha, USA; 6 Pain Management, NEMA Research, Inc., Naples, USA; 7 Pain Management, Paolo Procacci Foundation, Rome, ITA

**Keywords:** suicidality, suicide and depression, suicide, opioid overdose, mental health and suicide, suicidal intent, opioid epidemic

## Abstract

Opioid toxicity can result in life-threatening respiratory depression. Opioid-overdose mortality in the United States is high and increasing, but it is difficult to determine what proportion of those deaths might actually be suicides. The exact number of Americans who died of an opioid overdose but whose deaths might be classified as suicide remains unknown. It is important to differentiate between those who take opioids with the deliberate and unequivocal objective of committing suicide, that is, those with active intent, from those with passive intent. The passive-intent group understands the risks of opioid consumption and takes dangerous amounts, but with a more ambiguous attitude toward suicide. Thus, among decedents of opioid overdose, a large population dies by accident, whereas a small population dies intending to commit suicide; but between them exists a sub-population with equivocal intentions, waxing and waning between their desire to live and the carelessness about death. There may be a passive as well as active intent to commit suicide, but less is known about the passive motivation. It is important for public health efforts aimed at reducing both suicides and opioid-use disorder to better understand the range of motivations behind opioid-related suicides and how to combat them.

## Introduction and background

Opioid-induced respiratory depression is a potentially life-threatening consequence of opioid toxicity, but it is not always possible to determine if death from opioid overdose was accidental or intentional [1]. The Centers for Disease Control and Prevention (CDC) estimates that about 7% of all opioid overdose deaths are due to suicides [2]. But many opioid-associated deaths occur under ambiguous circumstances, most of which without a note or clear indication that would allow the death to be properly adjudicated [3]. Indeed, individuals rescued from a potentially fatal overdose may be unable or unwilling to explain their intentions [4].

For lives affected by opioid use disorder (OUD), mental processes and executive function may be impaired [5]. Learning processes that involve rewards are often overwhelmed by the opioids’ effect on neural pathways [5]. It is unclear how suicidal ideation forms in a person with OUD, particularly prolonged OUD, and whether and why such suicidal thoughts might persist in ways that could differ from those without exposure to opioids.

While it is well known that opioid use, as well as OUD, are associated with suicide [6], opioid overdose occurs across a full spectrum of people, from opioid-naïve individuals taking opioids for the first time in a clinically supervised situation, to those on chronic legitimate opioid prescriptions, to street drug users. In 2019, approximately 50,000 people in the United States died in an opioid-related overdose [7]. Our aim in doing a narrative review of the literature was to explore the nature of intent in opioid-associated suicides, as it appears that between the unequivocal intent to commit suicide and accidental overdose falls another category of more ambiguous intentionality. This number may be larger than we suspect, and it may represent a point at which public health interventions may be able to find a good “point of entry” in suicide prevention.

The authors searched the literature using the PubMed and Cochrane databases for the combination of “suicide” and “opioid” and obtained 1,378 and one results on PubMed and Cochrane databases, respectively. The articles were then sorted based on literature relevant to intention (n=47). The authors also used authoritative websites for data on opioid toxicity, opioid overdose, and suicide. The number of people who overdose on opioids in the United States has been reported and must be viewed as relatively reliable, but the number of people who commit suicide is not. While some suicides are clear, for instance, with the use of a “suicide note,” many deaths are ambiguous. It is likely that some suicides are deliberately obscured to protect the family from shame or other reasons. The authors sought to examine whether the high number of opioid overdose fatalities might be obscuring suicide deaths.

## Review

Suicidality and suicidal ideation are terms that clinicians understand and frequently use. However, these lack an expert consensus definition and reliable-objective metrics. Suicide is a major cause of death, and suicidal intentions are heterogeneous. It has been estimated that for every 31 people who admit having entertained thoughts of suicide, one will actually attempt suicide [8]. Suicidal ideation does not correlate well to suicide deaths. For instance, the highest rates of suicidal fatality in the United States occur in Caucasian men over the age of 75 (40 per 100,000 suicides), but this group has low rates of suicidal ideation; women over the age of 75 have higher rates of suicidal ideation, but ten-fold lower rates of fatality by suicide (four per 100,000) [8]. While opioid-related mortality is high [9], a subset of overdoses is intentional. Among the people attempting suicide using opioids, Gicquelais and colleagues draw a distinction between “active” and “passive” intent [10]. People with active intent are trying to die of suicide, while those with passive intent know the risks of death but simply do not care. Both groups understand the risks of opioid overdose. The active-intent group knowingly attempts suicide, while the passive-intent population has ambivalent attitudes about suicide and perhaps a sense of fatalism [10]. From a study of 274 residents in a treatment facility with a history of opioid overdose, 7% had used opioids with an active intent and deliberate purpose to commit suicide, but 44% reported they had used opioids with a passive intent to commit suicide [10].

An older study of individuals who commit suicide has named these groups “death seekers” (active-intent group) and “death darers” (passive-intent group) [11]. In many individuals who contemplate suicide, there is an ongoing internal conflict between an innate will to live and a desire to die, or at least, no longer be living [12,13]. Thus, barriers to suicide include a robust will to live, a fear of death, and lack of access to means of suicide. However, those taking opioids have at their disposal a ready means to commit suicide. Many people who take opioids have chronic pain, OUD, or both, any of which may expose them to overwhelming hardships and difficulties. Thus, those taking opioids have access to familiar tools to attempt suicide and perhaps less reticence than others to consider suicide.

The term “life weariness” has been connected to suicidal ideation. Life-weary people are those who find themselves emotionally ill-equipped for life, overwhelmed, depleted, marginalized, and thwarted in their efforts to move forward [14]. Life weariness may occur in older individuals or in those facing extreme challenges, such as a devastating illness or chronic pain. A study of 7,913 people over the age of 60 years living in Sweden found that those weary of life were more likely to live in urban or semi-urban areas, be older, be divorced, and have lower educational levels. Those both weary of life and suicidal were more likely to live in a residential care facility, be unmarried or widowed, have financial struggles, or were born in a non-Nordic European country (not part of the native-born population) [15]. A survey of 15,957 seniors in China found 17.7% of respondents had thought about suicide in the past year. Attributes related to suicidality were older age, financial concerns, dysfunction, depression, neglect by their children, limited social network, loneliness, and residing in an urban setting [16]. A survey of 15,105 Brazilian civil servants found that life weariness and suicidality were associated with stressful life events and a self-perception of poor physical health [17]. Growing older, in and of itself, may be associated with life weariness, as older individuals face diminished social and professional roles, failing health, and loss of control of their day-to-day life [18]. Life weariness has not been studied specifically in people with OUD, but living with OUD would seem to pose enormous challenges. Many problems of people with active substance use disorders seem to mirror those of life weariness: diminished social roles, failed relationships, unemployment or underemployment, health issues, and also legal and financial struggles. Thus, it may be that living with OUD over the long term, particularly for low-income individuals living in a street or urban setting, pre-disposes an individual to life weariness.

In many cases, suicide occurs because the individual seeks some sort of relief from an intolerable or untenable situation (e.g., physical and/or psychological pain) rather than desiring death as an endpoint. Psychological pain is not only real pain; it shares some neuronal pathways with physical pain [19]. OUD is associated with adverse childhood experiences, where users attempt to salve emotional pain with opioid use [20].

Most common mental health conditions have been associated with prescription and/or illicit opioid overdose [21] and suicide [22]. A study comparing intentional and unintentional overdoses found that those who overdosed accidentally were more likely to have strong substance use disorders, while those who overdosed deliberately were more likely to have mental health disorders [23]. 

The role of benzodiazepines in opioid-related suicide deserves to be considered. Benzodiazepines are among the most frequently prescribed drugs in the United States, and they are indicated for anxiety disorders or insomnia, among other conditions [24]. However, benzodiazepines are often prescribed off-label and often taken as chronic therapy and may be associated with physiologic dependence, making discontinuation of the drug difficult [25]. The CDC advised against the co-prescribing of opioids and benzodiazepines, as both are central nervous system depressants and together may increase the likelihood of respiratory depression [26]. Benzodiazepine monotherapy has been associated with suicidal ideation and completed suicide [27-29]. In a study of 3,465 suicide deaths in Colorado from 2015 to 2017, 60% had filled a prescription for a controlled substance during that time frame, and 14% filled a prescription for a benzodiazepine [28]. Twenty percent of all suicide deaths in this study (all methods, including firearms) had a recent exposure to benzodiazepines. Of those individuals who committed suicide and had a recent benzodiazepine exposure, half (50%) had filled a prescription for opioids in the past 30 days before death. Of those who died by suicide by drug overdose, 48% had recent benzodiazepine exposure [28]. It is not clear how many of these benzodiazepine-related suicides were carried out with active or passive intent.

Active suicidal ideation is similar to active suicidal intent. Active suicidal ideation means thinking about different ways to die and formulating a plan to carry it out. Passive suicidal intent is likewise similar to passive suicidal ideation, and it involves thoughts about no longer being alive or imagining being dead. A study of 153 chronic non-cancer pain patients found increased suicidal ideation with certain factors: higher scores of the Beck depression inventory, abdominal pain, and a family history of suicide attempts. Interestingly, the study did not associate suicidal ideation in these patients with the severity or duration of their pain or with their workman’s compensation status [30].

Suicidal intent is sometimes measured by premeditation, preparation, and the likelihood of rescue [31]. For instance, those who take steps to prevent discovery or intervention are considered more intentional than those who are more likely to be rescued [32]. Some suicide attempts are planned with care, but with the goal of getting rescued and thereby getting attention (the “cry for help”) as a form of extreme psychosocial communication [33]. In some cases, the individuals sought to use an attempted suicide to manipulate their environment or circumstances; and often had no further interest in suicide once their problem was addressed [34]. Thus, the intent of this population is not to commit suicide at all; but to use an attempt at self-harm as a means to an end. In fact, the individual’s response to being rescued from an attempted suicide is considered a good metric for future suicidality; rescued individuals who claim that they wish they had been left to die were two and a half times more likely to attempt suicide again than those who were happy or even ambivalent about survival [35]. However, it has been argued that attempted suicide and completed suicide are actually the same syndrome in the elderly population [36].

The more fixed and active suicidal intent is, the greater is the likelihood of the individual to die by suicide [37]. Furthermore, the greater and more focused the suicidal intent is, the more likely that the attempt will be medically lethal [38]. The clinical implications of opioid-associated overdoses being intentional or accidental are important to public health. There is likely a population of individuals who use opioids as a means to commit intentional suicide, and this group is not likely to respond to opioid treatment or rehabilitation efforts. There is a population of individuals who die of opioid-induced respiratory depression that occurs accidentally. Between these two groups is what may be a large population that is troubled, burdened, seemingly reckless, with little interest in many aspects of everyday living but ambivalent about dying. This group is rarely specifically addressed in public health efforts aimed at reducing suicide or combating OUD.

The role of opioids in suicide is multifaceted. On the one hand, opioids can induce an altered mental state, which may trigger suicidal ideation or make it harder for an individual to assess thoughts about death or dying rationally. On the other hand, opioids and mental health disorders are comorbid conditions, and opioids may relieve or serve to mask certain symptoms of anxiety or depression (“chemical coping”). Since most drugs address symptoms rather than root causes, it is possible that many people taking opioids have mental disorders associated with suicidality that may be only unreliably relieved by their opioid regimen, and it is these disorders that prompt suicidal tendencies.

Reducing opioid overdose deaths requires a more nuanced understanding of them. For certain individuals, opioids are a tool, but if opioids are not available, they may choose firearms or some other means [39]. Cognitive behavioral therapy or other types of psychological counseling such as motivational interviewing may be beneficial in reducing suicide risk [40].

The passive-intent group is far more difficult to quantify and to treat. These individuals may have OUD or some other form of substance-use disorder that permits them to regularly test the boundaries between tolerated and potentially dangerous doses. Patients with current substance use disorders should be given care with respect to suicide prevention as well as harm reduction [21,41]. It may be beneficial to screen such patients for mental health conditions that might play a role in suicidal ideation, particularly depression [42]. Tools to help stratify patient risk have been developed [43-45]. Substance use disorder programs, rehabilitation centers, addiction specialists, and others may offer comprehensive programs, but it may be that primary care providers or community centers can offer important care as well that may be more accessible to certain individuals [46].

Finally, it is important to mention that many people take prescription opioids to manage persistent moderate to severe pain. Chronic pain is in itself associated with suicide, apart from OUD or mental health disorders [47]. While chronic pain may cause some individuals to become hopeless and thus suicidal [48], there are neurological links between pain and suicidality [49]. Chronic pain may induce a persistent pro-inflammatory state that has been linked to suicide; dysregulation of the immune system has also been associated with suicide [50]. Thus, in chronic pain patients, suicide by opioids may have a deeper and more complex etiology.

This is a narrative review with its inherent limitations. It is not a systematic review, and the subject of the degrees of intention for committing suicide by opioid defies easy quantification. The literature is sometimes old and has methodological concerns related to the complex topic. Nevertheless, it is an important subject worthy of further deliberation.

## Conclusions

The role of intention in suicide-by-opioid is an important subject because it may affect how health care providers and public health experts can best address the alarming trend of deliberate death using opioids. Those with active intent take opioids as a means to an end and have a clear desire to commit suicide. This population should be treated with tools to address suicidality. Those with a passive intent take opioids recklessly, knowing that the dangerous doses they consume may kill them, but do so with ambivalence about the possibility that the dose may be fatal. This group is likely larger than the active-intent group and is more challenging to treat because they typically have OUD or some other form of substance use disorder and require interventions to control their use disorder in addition to their suicidality. Further study is needed, particularly to better understand and thus find targeted interventions for the passive-intent group.
